# Study on segmentation of blasting fragment images from open-pit mine based on U-CARFnet

**DOI:** 10.1371/journal.pone.0291115

**Published:** 2023-09-14

**Authors:** Changyu Jin, Junyu Liang, Chunhui Fan, Lijun Chen, Qiang Wang, Yu Lu, Kai Wang

**Affiliations:** 1 Key Laboratory of Ministry of Education on Safe Mining of Deep Metal Mines, Northeastern University, Shenyang, 110819, China; 2 China Railway 19th Bureau Group Mining Investment Co., Ltd., Beijing, 100161, China; University of Alcalá, SPAIN

## Abstract

Bench blasting is the primary means of production in open-pit metal mines. The size of the resulting rock mass after blasting has a significant impact on production cost. Currently, the ore fragment size is obtained mainly through manual measurement or estimation with the naked eye, which is inefficient and inaccurate. This study proposes the U-CARFnet and U-Net models for segmenting blasting fragment images from open-pit mines based on an attention mechanism, residual learning module, and focal loss function. It compares this technique with traditional image segmentation ones and a variety of deep learning models to verify the efficacy of the proposed model. Experimental results show that the accuracy of the U-CARFnet model proposed in this paper reaches 97.11% in the performance evaluation, which shows better performance than the traditional image segmentation method. In this study, the U-CARFnet model is used in the application, and a superior performance is obtained, with an average segmentation error of 5.46%. The proposed approach provides an effective technique for statistically analyzing images of mine rock.

## 1. Introduction

The size distribution of blasting fragments and the percentage of large ore blocks are crucial indices for evaluating the blasting effect in open-pit mines. The accuracy of statistical analysis of ore fragments is vital to mine production efficiency and blasting design. Currently, the ore fragment size is determined by manual measurement or estimation using the naked eye in most open-pit mines. Due to the bench height, the blasting fragments generated by a single blast can cover a large area. Manual evaluation of blasting fragment distribution is time-consuming, inefficient, and inaccurate. However, severe potential safety hazards exist during long-time measurements of blasting fragments. As a result of the rapid growth of computer software, image processing technology has made significant strides and is now widely applied in mining engineering. Regarding image processing for blasting fragments, various image segmentation methods, including watershed (Wang et al. [1]), threshold segmentation (Lu et al. [2]), and other modified methods (Dong and Jiang [3]; Chalfoun et al. [4]), have been proposed by experts and scholars. In general, there are often issues with the images of blasting fragments from open-pit mines, such as shadow overlap caused by uneven illumination, unclear contours because of soil particles on the surfaces of blasting fragments, and ore fragment adhesion and accumulation due to deposits in between. As a result, accurate image information cannot be acquired using the mentioned image processing approaches; therefore, it is difficult to provide accurate data for subsequent image recognition.

Due to the rapid development of the deep learning technique, it has been applied in various fields because of its strong ability to learn specific features from a large amount of training data. Navab et al. [5] first proposed the U-Net network with an encoder-decoder structure, which was applied to segment cell images and realized high-precision target segmentation with a small number of cells. Raza et al. [6], Oktay et al. [7], and Sevastopolsky et al. [8] successfully used the same technique in other medical fields.

The applications of convolutional neural network (CNN) based on deep learning models have been extensively investigated in medical image segmentation. Due to the similarity between ore segmentation in geotechnical engineering and organ segmentation (especially cell segmentation), ore image segmentation and recognition research has been encouraged. The traditional algorithms have difficulty distinguishing targets from the background in ore images for mineral particle size analysis in complex scenes. Zhan and Zhang [9] proposed an ore segmentation method using histogram cumulative moment and applied it to ore positioning and recognition in multi-scenario. Meanwhile, the traditional U-Net model suggested the U-Net-based transformer technique in light of learning information loss during ore image segmentation. (Xiao et al. [10]) presented the RDU-Net model to determine the size of ore fragments on the conveyor belt. The performance of the DUNet model in ore image segmentation was superior to that of U-Net. (Duan et al. [11]) designed a lightweight U-Net deep learning network for detecting and segmenting iron ore green pellets in images. The particle sizes of green pellets were determined by separating pellets with the concentric circle model and detecting pellet shape with ellipse fitting. Olivier et al. [12] proposed the VGG16 model for estimating ore particle size distribution. Mustafa et al. [13] conducted iron ore region segmentation using high-resolution remote sensing images based on Res-U-Net. (X. Liu et al. [14]) introduced a deep learning U-Net model for image segmentation, and the outcomes were binarized and input into the Res-Unet convolutional network to achieve the final segmentation results, which were illustrated by OpenCV. They addressed the issue of low segmentation accuracy caused by the adhesion of ore particles and dark areas in the images. (H. Yang et al. [15]) proposed an improved U-Net for ore image segmentation with VGG-16 as the encoder. (Y. Liu et al. [16]) discussed an efficient segmentation model based on the adhesion and overlap between ore particles by splitting and integrating five classical deep learning segmentation models, comparing their segmentation performance results, and introducing a new loss function. The study was also expanded to include semantic segmentation of microscope images. Filippo et al. [17] conducted investigations on semantic segmentation of opaque and non-opaque minerals from epoxy resin in reflected light microscopy images using the DeepLabv3+ model. Yang et al. [18] analyzed the particle size distribution of fragmented rock based on superpixel image segmentation. Koh et al. [19] compared the performance of two types of segmentation algorithms, Mask R-CNN and SOLO v2, to overcome the limitations of thin-section microscopy; hence, identify grain boundaries and classify minerals more rapidly. Lu et al. [20] used color image recognition to segment materials on a conveyor belt.

This study proposes a new image segmentation model, U-CARFnet, based on the attention mechanism, the residual learning module, and the focal loss function, as a result of the information loss in the fragment size distribution from bench blasting caused by conventional image segmentation techniques. The proposed model has good image segmentation performance. It can solve the difficulties in obtaining the blasting fragmentation information in open-pit mines and provide efficient and accurate data support for predicting blasting fragmentation and optimizing blasting parameters.

## 2. Project overview

### 2.1 Geological conditions in the mining area

**Fig 1:**
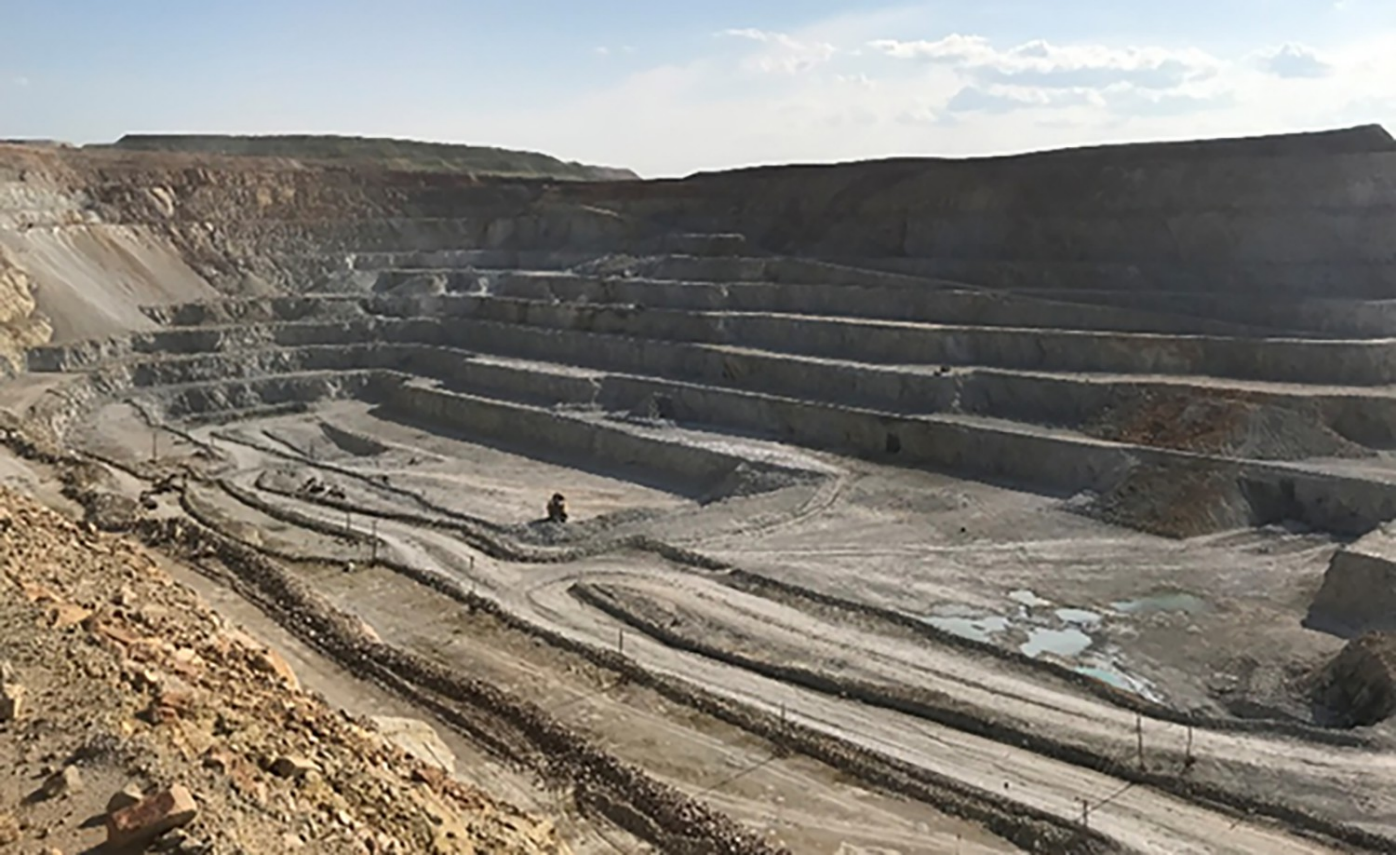
Panoramic view of the open-pit mine.

This study considers a copper-molybdenum mine in New Balhu Right Banner, Manzhouli, Inner Mongolia Autonomous Region, China, as a test area. The geographical coordinates of the copper-molybdenum mine are 117° 15′ E − 117° 20′ E in longitude and 49° 24′ N − 49° 26′ 30″ N in latitude. The deposit is a massive continental subvolcanic porphyry copper-molybdenum deposit controlled by volcanic mechanisms. The ore belt is long-ring shaped, with a long axis length of 2600 m and a short axis length of 1350 m. Its strike is approximately 50° and generally dips toward the northwest. The dipping angle changes gradually from 85° to 75° from east to west. The depth of the deposit is greater than EL200. The mining depth is 600 m below the earth’s surface, and the average thickness of the ore body is about 260 m. The inner ring of the ore body is mainly molybdenum, and copper dominates the outer ring. Fig 1 demonstrates the panoramic view of the copper-molybdenum open-pit mine.

### 2.2 Physical properties of ore rock

**Fig 2:**
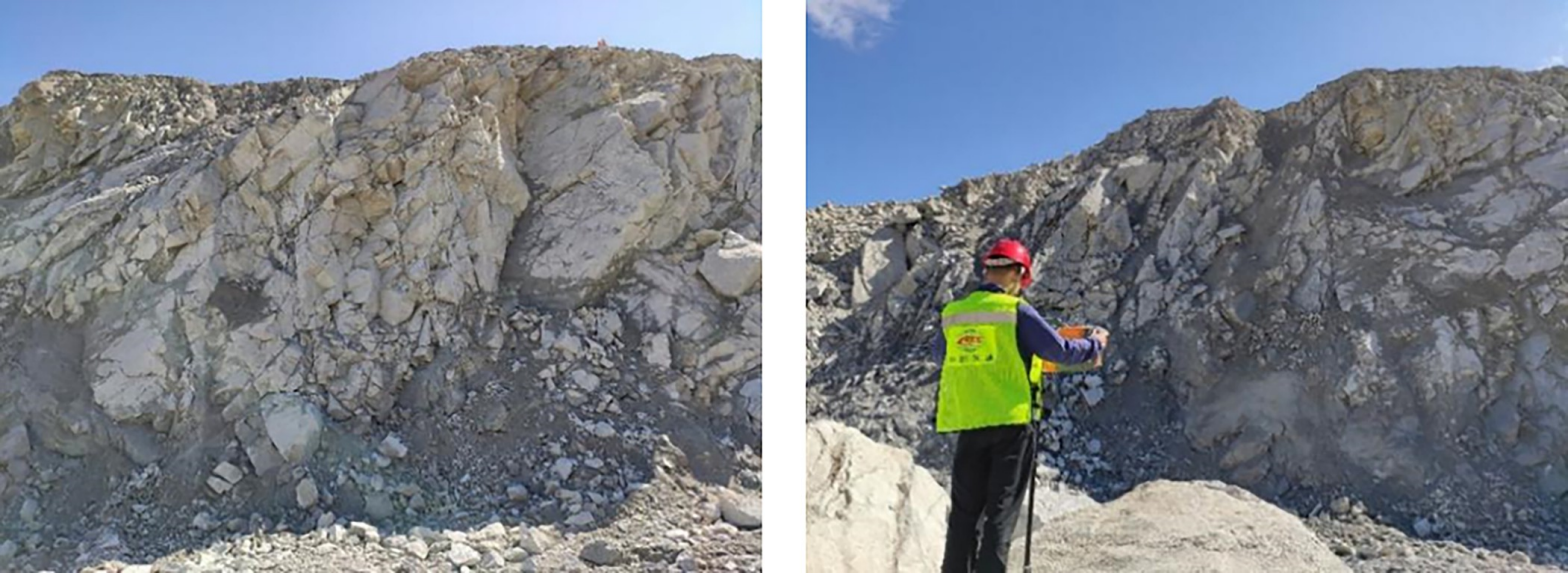
Bench slope of open-pit mine.

Based on the previous geological and deposit data in the study area, the rock outcrop exposed in the Wushan mining area mainly includes biotite granite, granodiorite (vein), quartz monzonite porphyry, diabase porphyrite, dacite porphyrite, andesite porphyrite, rhyolitic porphyry (or felsic), tectonobrecia (rhyolitic and granitic), volcanic breccia, detonated breccia and propylite. The main metal elements in the deposit are Cu and Mo. Granite is the rock with the largest outcrop area in the mining location. The rock masses have a distinct strike direction and contain many fissures. Fig 2 depicts the bench slopes in the open-pit mine.

### 2.3 Field bench blasting effect

**Fig 3:**
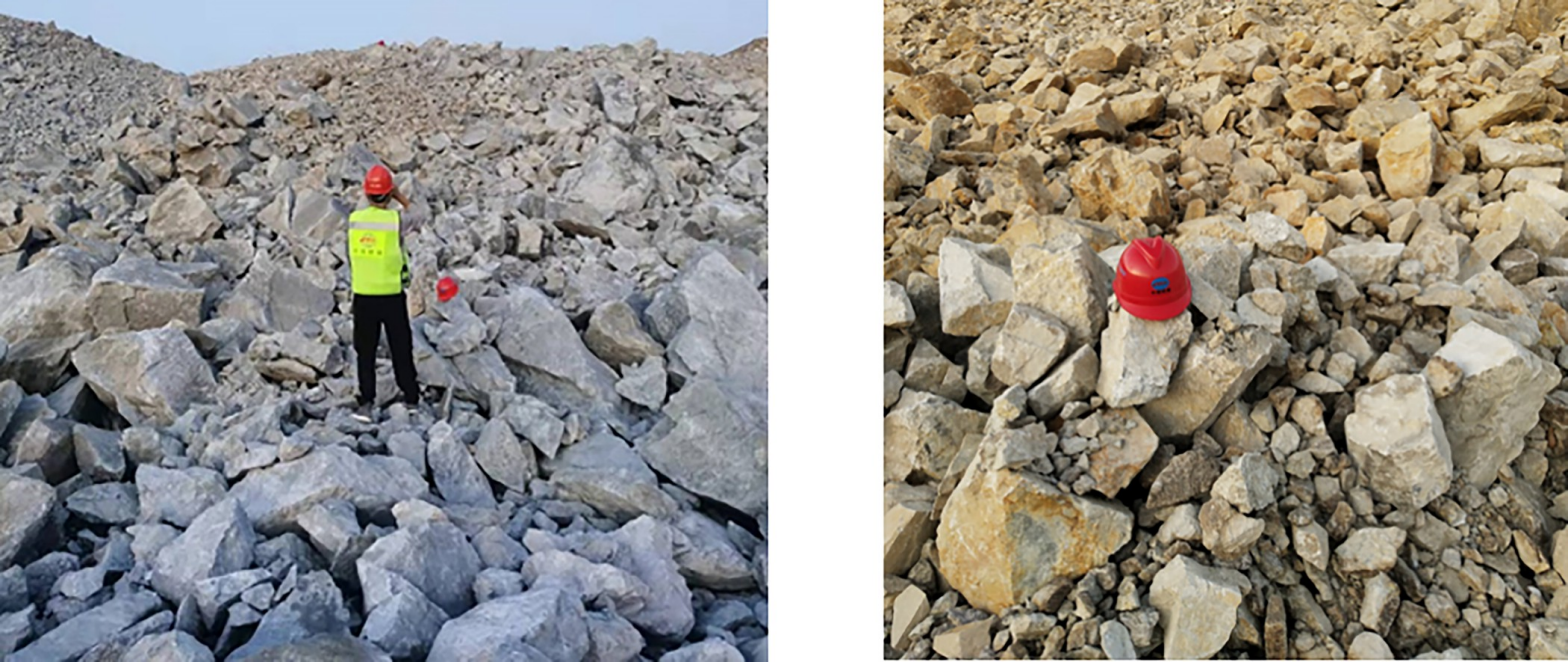
Blasting fragments on the site.

As the rock masses in the mining area are relatively hard and intersected by several groups of primary fractures, the percentage of large rock blocks after blasting has been one of the most significant technical challenges encountered during mining production in Wushan Copper Mine. In addition, although multiple groups of primary fractures intersect both the east and west mining areas, the bench blasting effects are quite different. More large rock blocks are produced in the east mining area. Hence, the study on blasting fragment distribution in the site is significant for optimizing blasting parameters, improving the blasting impact, and reducing large block percentage. Fig 3 illustrates the rock fragments after blasting on the site.

## 3. Data acquisition and processing

### 3.1 Image acquisition for blasting fragments

**Fig 4:**
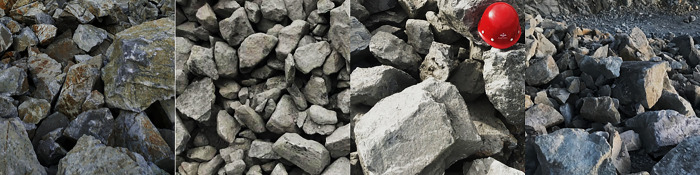
Images of blasting fragments.

Deep learning relies heavily on experimental data as an essential foundation for network training and learning. After bench blasting on the site, images were collected for the fragments to capture many valid ore image datasets. The datasets used in this study were collected using mobile phones (Fig 4). The mobile phone model and configuration is a Vivo X21, which is equipped with a rear-facing 2x12-megapixel (24-megapixel photosensitive elements) primary camera and a 5-megapixel secondary camera.

### 3.2 Dataset processing for blasting fragment images

**Fig 5:**
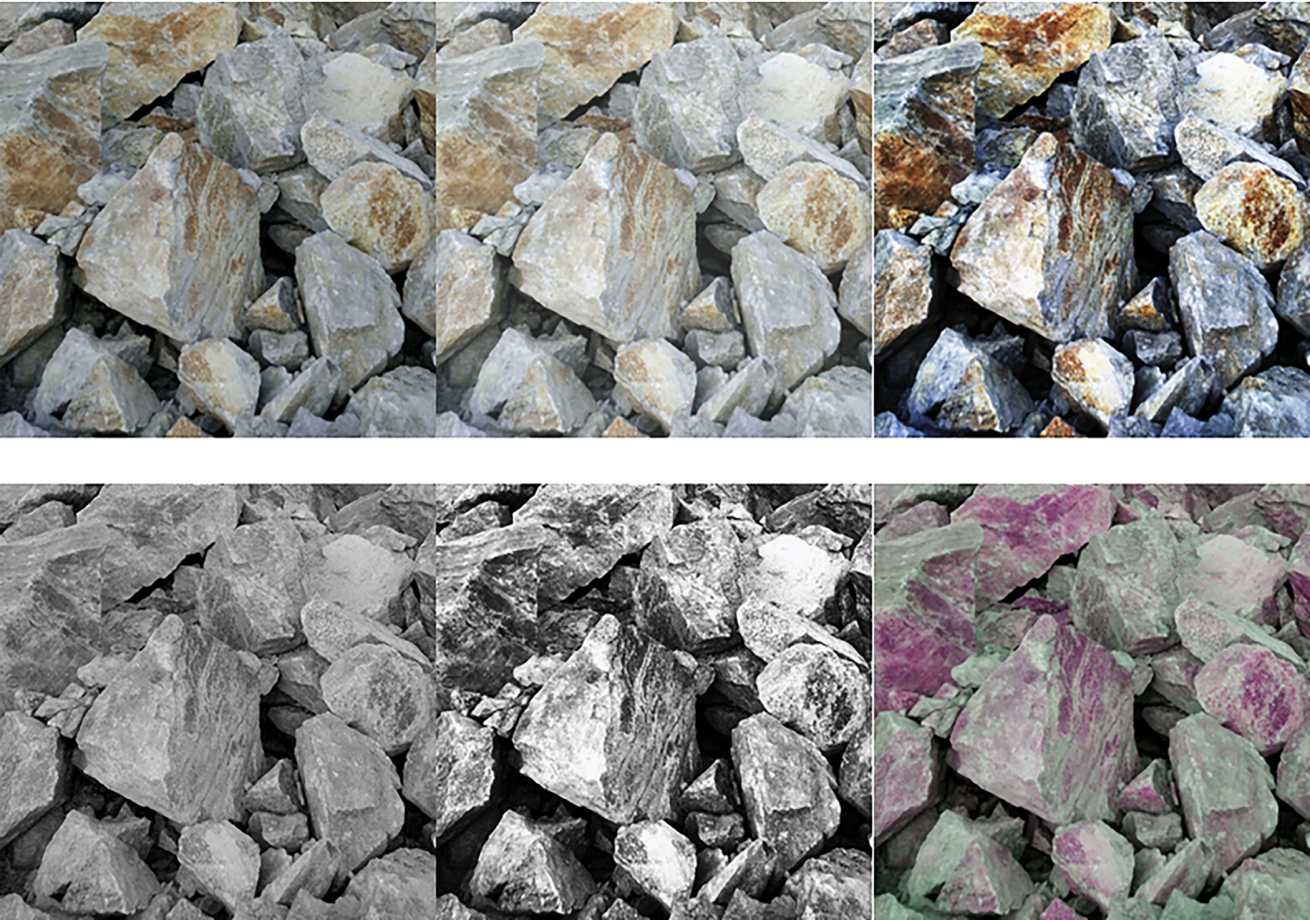
Pre-processing of ore fragment image.

Fig 5 indicates that the image data are pre-processed by adding random pixels, histogram equalization, gray processing, gray+histogram equalization, and HSV transformation to obtain more training data and enhanced training performance. Finally, the noise in the pre-processed image is reduced to produce an expanded dataset.

**Fig 6:**
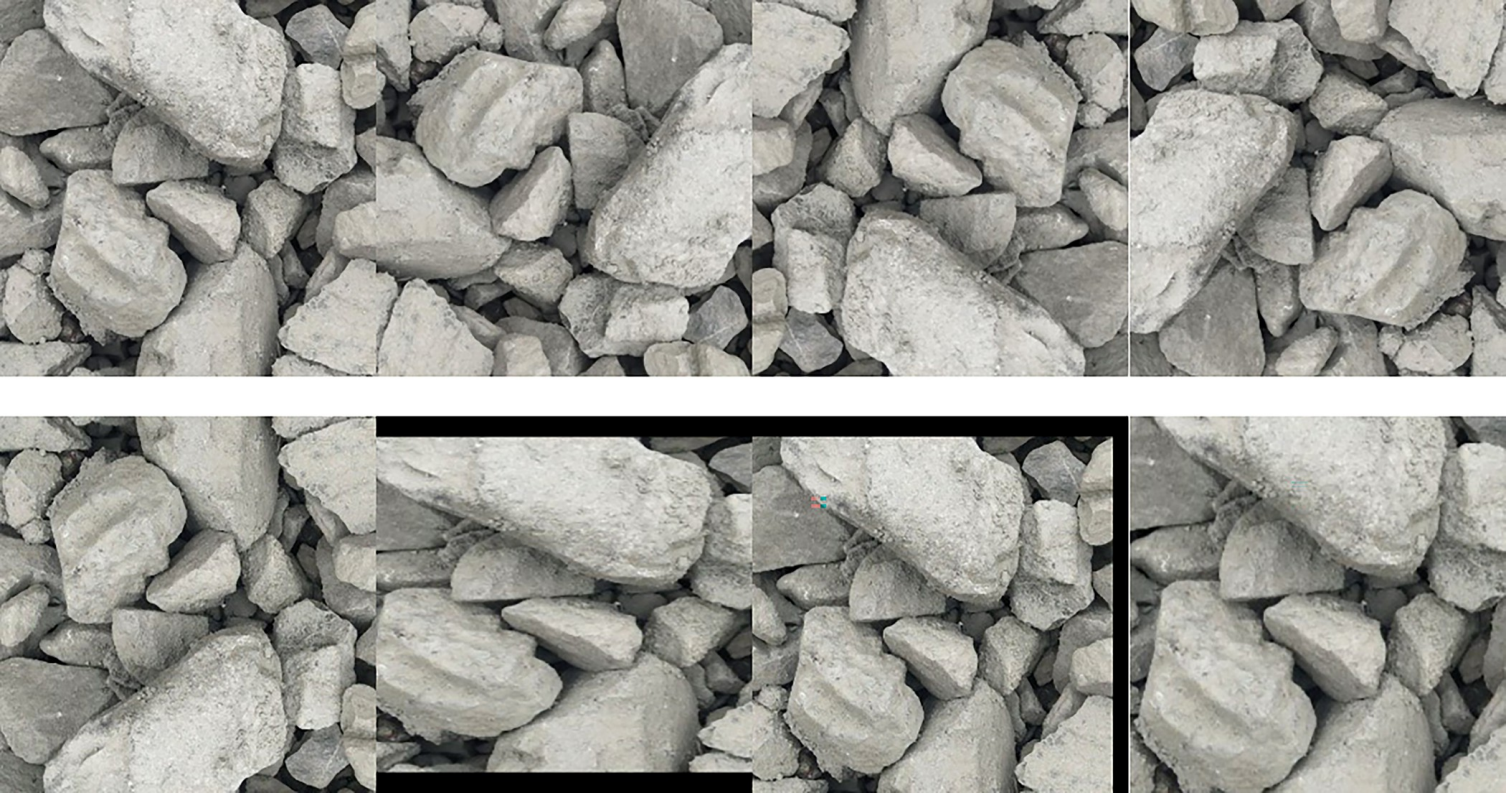
Images after rotation, flipping, and scaling.

Rotation (90°, 180°, 270°), flipping (horizontally and vertically), and scaling (equal proportion or unequal proportion) are performed on the expanded dataset to increase the quantity of data, as revealed in Fig 6. Finally, a dataset with 23168 512 ×512 pixel images is acquired, which is 64 times of the original dataset.

### 3.3 Dataset preparation for blasting fragment images

**Fig 7:**
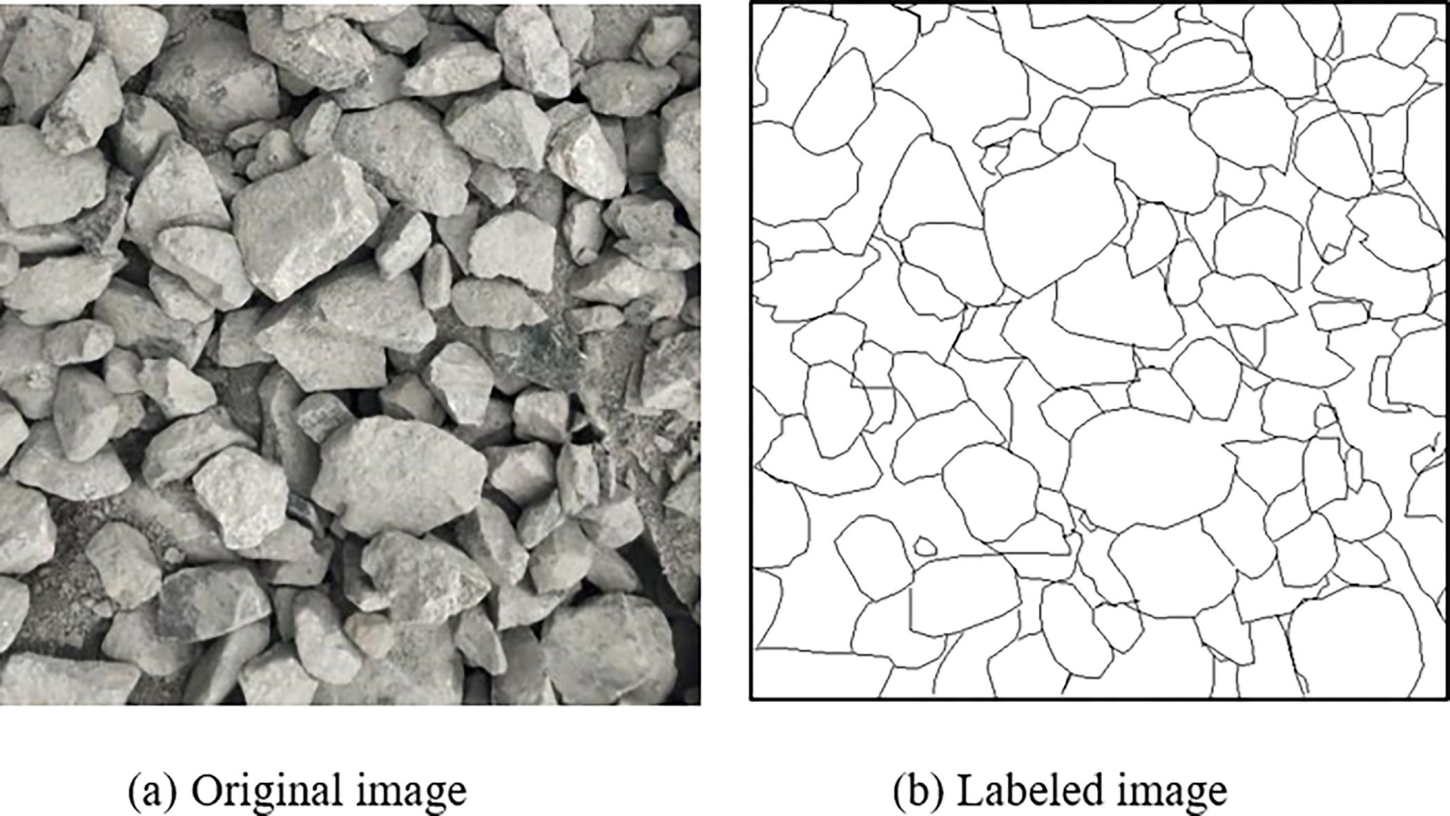
Labeling of image contour.

In order to meet the requirements of convolutional neural network training, the blasting fragment images must be cut and labeled based on a unified size to obtain the input data for the image segmentation network. The ore rock contours are manually labeled using an image labeling tool, "Labelme". The labels are distinguished by color; for instance, the white part represents the internal information of the ore rock, while the black line indicates the contour of the ore rock, as exhibited in Fig 7. In reality, the problem investigated by the generated training dataset is binary classification.

## 4. U-CARFnet model development

### 4.1 Attention mechanism

The vision attention mechanism is a unique signal-processing approach for human vision. Human beings quickly scan the global image through the eyes and obtain the target area where attention shall be paid, i.e., the focus of attention. Thereafter, more attention is paid to the target area to obtain more detailed information about the target with attention, while other useless information is suppressed. The attention mechanism in deep learning is essentially similar to human beings’ selective vision attention mechanism. The core goal herein is also to select the most critical information in a certain task from a large amount of information. Accordingly, the attention mechanism is an approach for realizing the adaptive attention of a network. Generally speaking, the attention mechanism can be divided into a channel, spatial, and a combination of the two mechanisms. Three attention mechanisms, namely, SENet (Hu et al. [21]), CBAM (Woo et al. [22]), and ECANet (Wang et al. [23]), are investigated in this paper.

**Fig 8:**
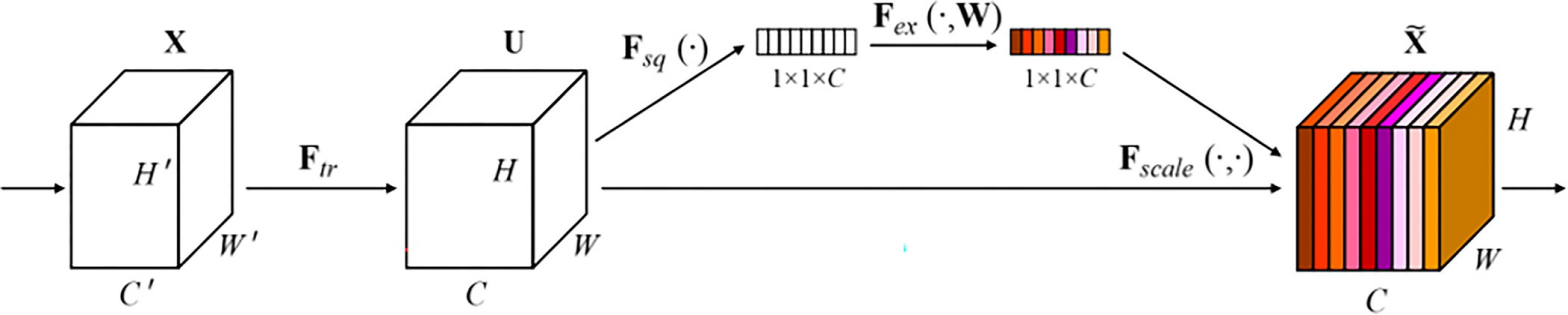
Squeeze-and-excitation block.

SENet (Fig 8) is a model that implements the channel attention mechanism. This model intends to obtain the weight of each channel in the input feature layer. With the help of SENet, attention is mainly paid to the channel that the network most requires. During the implementation of this model, the global average pooling is performed for the input feature layer before making the two full connections. The number of neurons in the first full connection is set to a small value, while the number of neurons in the second full connection is set as that of the input feature layer. Any sigmoid value between 0 and 1 is retaken after the two full connections. A weight (between 0 and 1) is assigned to each channel of the input feature layer, which is then multiplied by the original input feature layer. On this basis, learning the image features using SENet is implemented.

**Fig 9:**
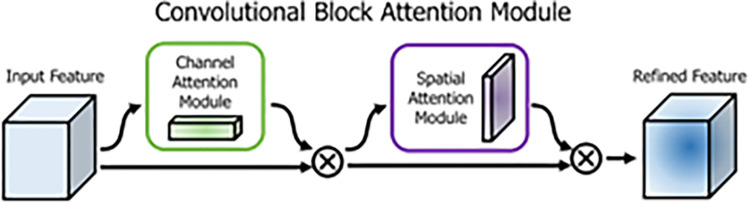
Illustration of the CBAM model.

CBAM (Fig 9) integrates the channel and spatial attention mechanisms. First, the global average pooling and global max pooling are performed for each input feature layer by the channel attention mechanism. The shared full-connection layer then processes the pooling results. After that, the processed results are added, and a sigmoid value is taken. In this way, a weight (between 0 to 1) for each channel of the input feature layer is obtained and then multiplied by the original input feature layer. The maximum and average values for each feature point on the channel in each input feature layer are calculated. Thereafter, the two values are stacked, and the number of channels is adjusted by their convolution, with the channel number being 1. Another sigmoid value is taken, and each feature point’s weight (between 0 to 1) is obtained in the input feature layer. The weight is then multiplied by the original input feature layer. In the CBAM, more valid features can be learned through the channel and spatial attention mechanisms.

**Fig 10:**
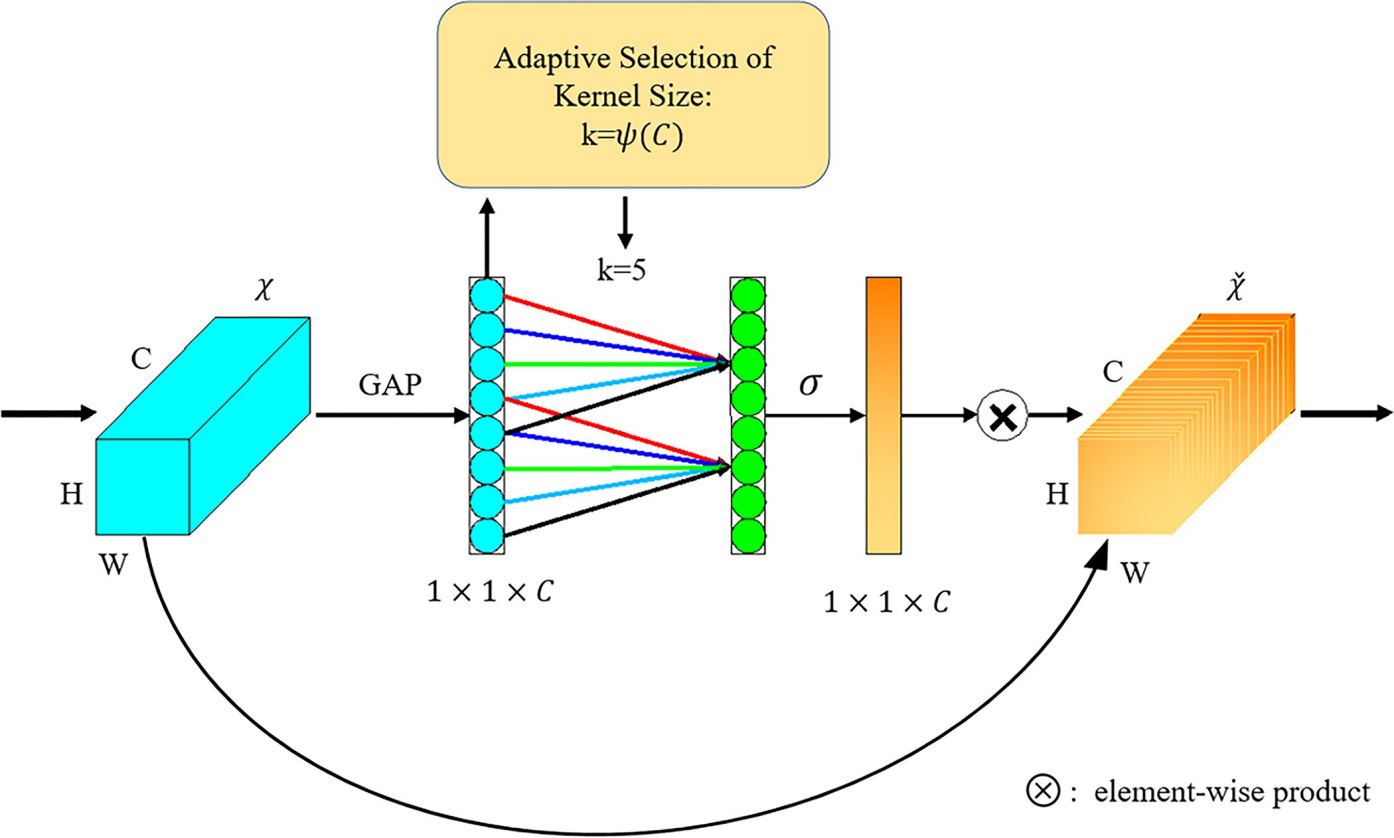
Efficient Channel Attention (ECA) module.

ECANet (Fig 10) is an implementation form of the channel attention mechanism and is regarded as an improved version of the SENet. The idea of the ECA module is straightforward, such that it removes the full-connection layers in the SE module and conducts learning by 1D convolution of features after global average pooling directly so that the convolution can obtain cross-channel information.

The attention module can significantly enhance the feature learning ability of convolutional neural networks. Hence, the effect of ore image segmentation based on the attention module integrated into the U-Net model is investigated in the paper.

### 4.2 Residual learning module

During the convolutional neural network learning process, increasing the depth of network layers results in more information being missed or ignored and longer training time being required. Indeed, residual learning emerged after proposing the ResNet model (He et al. [24]). Generally, the application of residual learning modules can, to a certain extent, solve information loss problems during transmission by traditional convolutional networks or fully-connected networks. A direct channel is added in the ResNet to allow the original input information to be directly passed to the deeper layer to protect the information’s integrity and simplify the learning targets and difficulties. The structure of ResNet can accelerate the training of neural networks and greatly enhance the model’s accuracy.

### 4.3 U-Net model and focal loss function

Unlike general convolutional neural networks, the U-Net model is a semantic segmentation network that relies on expanding and modifying a fully convolutional network (Navab et al. [5]). The network comprises a contracting path to obtain context information (down-sampling) and a symmetrical expanding path for an accurate position (up-sampling). Feature extraction is carried out through down-sampling, such as the location, semantics, and other image information. The abstract features are then restored and decoded to the size of the original image through up-sampling. Finally, the image is segmented at the pixel level.

The problem under study is a binary classification problem. In the traditional U-Net model, the binary cross entropy loss function, binary_crossentropy, is used to segment the cell images. In this study, due to the severe deviation between the ore contour information and the background in the labeled dataset of blasting fragment images, the binary cross entropy loss function, binary_crossentropy, may lead to reduced learning accuracy due to uneven proportion of positive and negative samples. The focal loss function is a cross-entropy with a weighted alpha to balance the uneven proportion of the positive and negative samples and solve the imbalance between easy and hard samples (Lin et al. [25]). Therefore, in this study, the focal loss function is adopted in the network to optimize the training performance under severe class imbalance.

The binary cross entropy loss function, binary_crossentropy:

$$CE(p,y)=-y\;\log\;p-(1-y)\log(1-p)\{\begin{array}{l} \;-\log\left(p\right)\;y=1\\ -\log\left(1-p\right)\;otherwise \end{array}$$ (1)

$$p_{t}=\left\{ \begin{array}{l} \;p\;if\;y=1\\ 1-p\;otherwise \end{array}\right.$$ (2)

$$CE(p,y)=CE(p_{t})=-\log\left(p_{t}\right)$$ (3)

where p is the test result, and y is the actual label.

It can be seen from the above equations that, with an ordinary cross-entropy, the higher the output probability, the smaller the loss for positive samples, and the lower the output probability, the smaller the loss for negative samples. The loss function is relatively slow in the iterative process for many simple samples and may not be optimized to the best. The focal loss function can solve such problems.

$$CE(p_{t})=-\alpha_{t}\log\left(p_{t}\right)$$ (4)

$$FL(p_{t})=-{\left(1-p_{t}\right)}^{\gamma }\log\left(p_{t}\right)$$ (5)

$$FL(p_{t})=-\alpha_{t}{\left(1-p_{t}\right)}^{\gamma }\log\left(p_{t}\right)$$ (6)

where *CE*(*p*_*t*_) can solve the class imbalance, while *FL*(*p*_*t*_) can solve the imbalance between easy and hard samples.

Finally, the two separate improvements are combined to form the focal loss function in the form of *FL*(*p*_*t*_). This solves the imbalance between positive and negative samples and between the easy and hard samples.

### 4.4 U-CARFnet model

By integrating the features of the U-Net model, attention mechanism, and residual learning module, the idea of the channel and spatial attention mechanisms and the skip connection are introduced into the U-Net structure to develop a new model structure called the U-CARFnet. With the U-Net model as the backbone, the residual learning module and ResNet are introduced in the convolution process to form the ResU-Net model framework. The attention mechanism is added in the up-sampling process, and finally, the focal loss function is applied. The model can optimize the convolutional learning process, enhance the feature extraction rate of learning targets, effectively prevent information loss in the process of information transmission between deep convolution layers, and significantly improve the model accuracy in learning.

**Fig 11:**
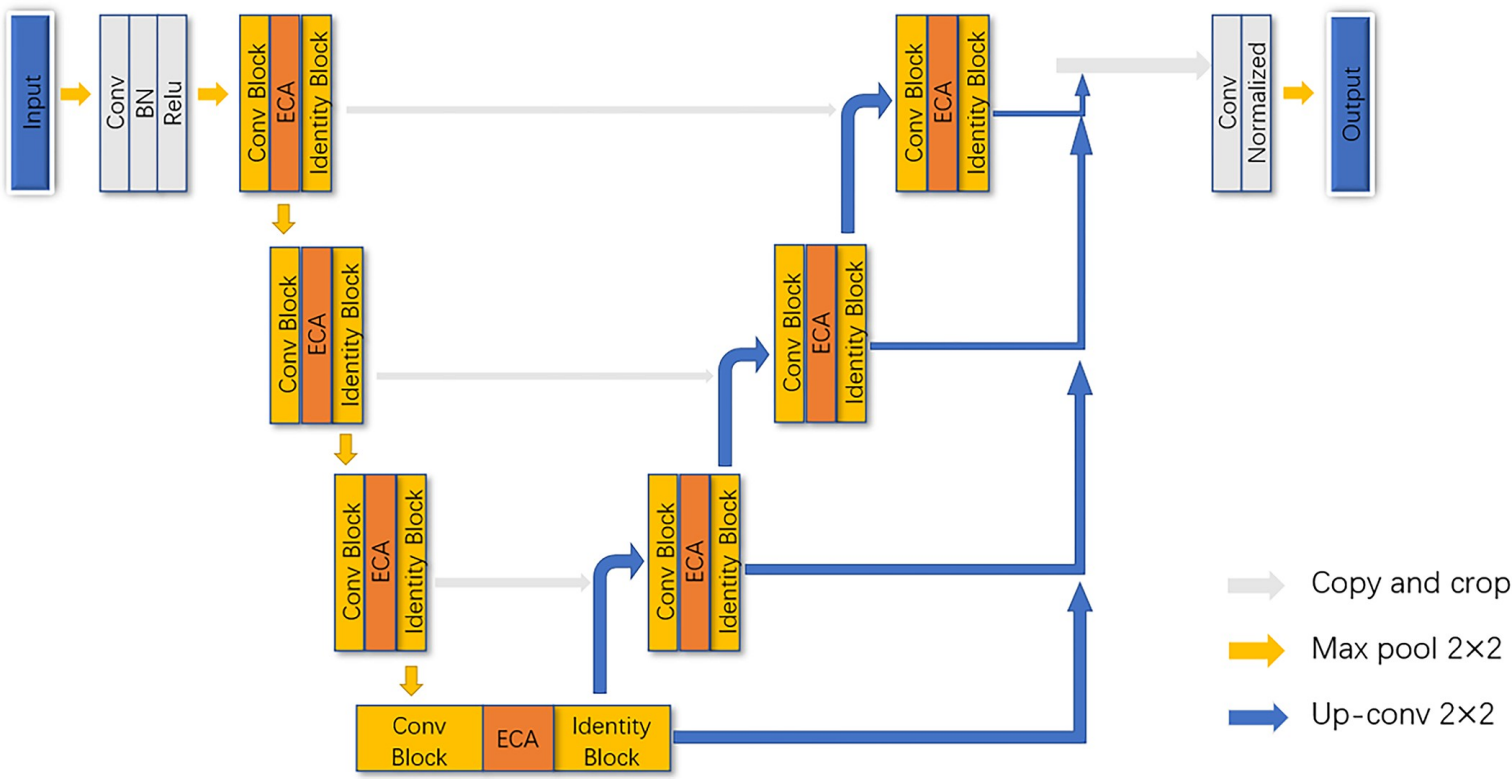
Structure of U-CARFnet model.

The model adopts a U-shaped network structure, utilizes the learning ability of skip connection in the residual learning module, extracts the context information in combination with the lower-level and higher-level features, and reduces the learning information loss between layers during the transmission process. As the attention learning module is added in each up-sampling process, the labeled contour information of the ore rock can be captured to develop accurate contour segmentation of the blasting fragments. Generally, more information can be learned using the focal loss function even under severe class imbalance in binary classification problems involving ore contour and background in the blasting fragment images. The false information learned by the convolution layers in case of severe class imbalance is optimized. Fig 11 illustrates the overall structure of U-CARFnet.

**Fig 12:**
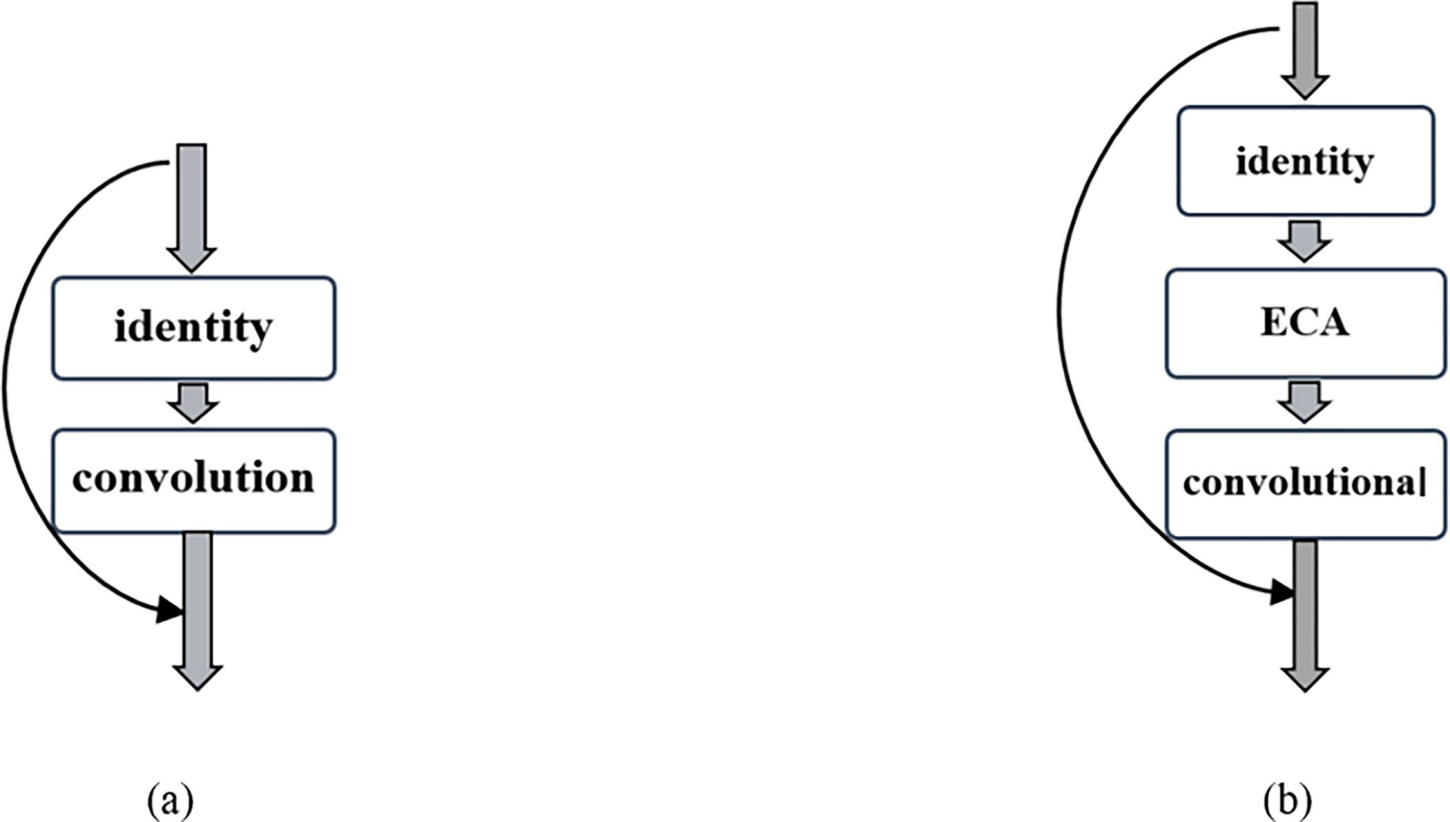
Learning module. (a) original residual learning module, (b) CEIB learning module with integrated attention model.

Fig 12 shows the residual learning module and the CEIB learning module modified from the deformable convolutional block integrated with the attention mechanism in the U-CARFnet model. In general, the deformable convolutional block can locally perceive each feature in the image using the convolution layer. The local features are integrated at a high level to extract the global feature information. The learning ability of skip connection in the residual learning module is then utilized to transmit the low-level and high-level information to the next stage. The features are extracted using the next group of the convolution operation. Finally, the attention learning module is introduced to optimize the information learning and extraction ability and solve the problem of information error and loss during learning and transmission.

## 5. Results and discussions

### 5.1 Model training

In order to perform the experimental study on the learning network model for blasting fragment image segmentation in the paper, a Server computer with Intel(R) Core (TM) i7 processor, MSI PTX 3070 GPU graphics card, and Windows 10 64-bit operating system was utilized. The configuration of the training environment is Tensorflow 2.4.0. A GPU training mode combined with CUDA 8.0.5.39 and CUDA 11.0 was employed to accelerate the training and learning processes of the model.

Before inputting the data into the model for training, the processed dataset was normalized, and the size was unified to obtain standard unified size information. Additionally, the blasting fragment images taken at various regions were mixed to avoid the impact of the dataset on model training, hence achieving improved learning performance. The dataset was divided into three parts in the model training process: 80% training dataset, 20% testing dataset, and the unlabeled blasting fragment images as the validation dataset.

### 5.2 Evaluation of model performance

#### 5.2.1 Evaluation metrics

In order to evaluate the training performance of the model, various evaluation metrics, including the accuracy (ACC), the area under curve (AUC), the positive predictive value (PPV), and the true positive rate (TPR), are adopted in this study.

The ACC refers to the degree of matching between the average value after multiple measurements under a certain experimental condition and the true value. Generally, it indicates the ratio between the number of true predictive samples and the total number of samples. The AUC is the area under the ROC curve (TPR versus FPR). It is used for binary classification problems. Generally, it can be intuitively explained as the probability that the predicted value of a randomly-chosen positive sample is greater than that of a randomly-chosen negative sample. The PPV, the precision, calculates the probability of the true positive samples in the outcomes. The TPR is the sensitivity or the recall rate, which indicates the ratio between the number of true positive samples and the total number of positive samples, i.e., the proportion of accurate predictions for the positive samples. These indices are utilized to evaluate the model’s performance. The formulas of these indices are as follows:

$$ACC=\frac{\left(TP+TN\right)}{\left(TP+FN+FP+TN\right)}$$ (7)

$$AUC=\frac{\sum_{i}^{P*N}I({score}_{pos}>{score}_{neg})}{P*N}$$ (8)

$$PPV=\frac{TP}{\left(TP+FN+FP+TN\right)}$$ (9)

$$TPR=\frac{TP}{TP+FN}$$ (10)

where TP (True Positive) is the number of positive samples (actually True) predicted by the model; FN (False Negative) is the number of negative (actually True) samples predicted by the model; FP (False Positive) is the number of positive samples (actually False) predicted by the model; TN (True Negative) is the number of negative samples (actually False) predicted by the model; P is the number of positive samples; N is the number of negative samples; *score*_*pos*_ is the score of positive samples; *score*_*neg*_ is the score of negative samples.

#### 5.2.2 Segmentation performance of models with different attention modules

**Fig 13:**
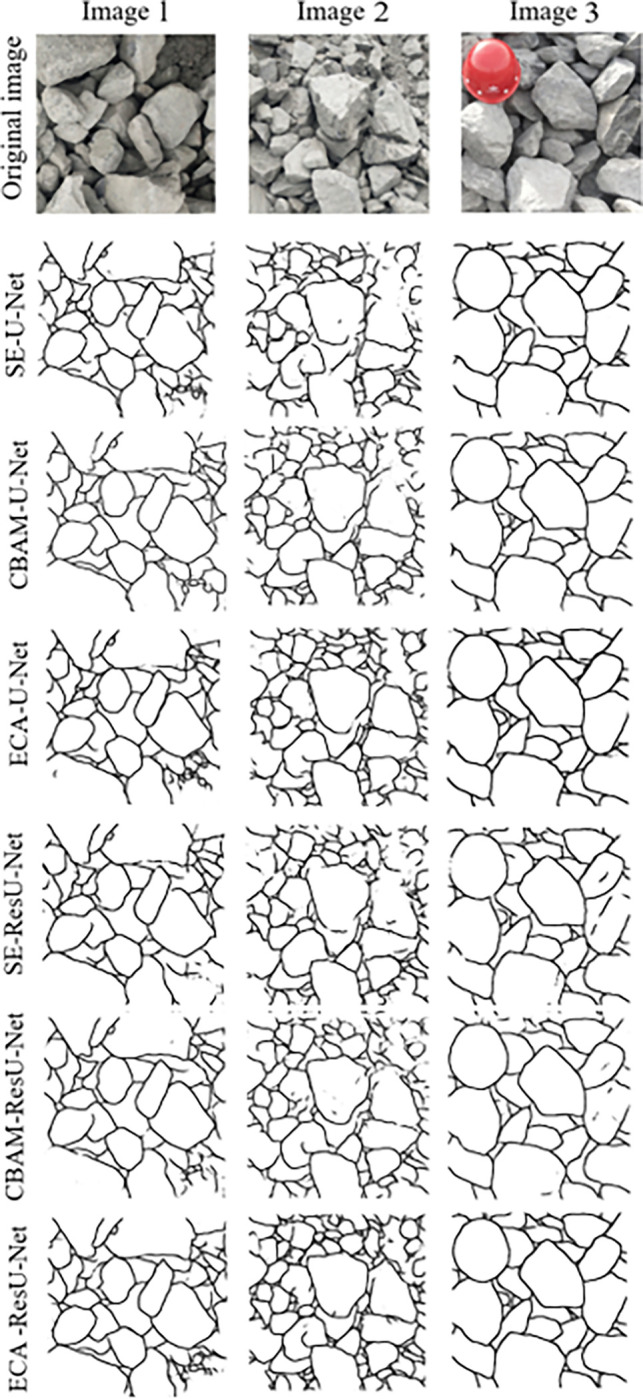
Comparison of segmentation performance by modified models.

In this study, the attention module is studied as a variable to achieve good segmentation performance for blasting fragment images. The SE-U-Net model with the channel attention mechanism SENet added after each U-Net convolution layer, the CBAM-U-Net model with CBAM added after each U-net convolution layer, the ECA-U-Net model with ECA mechanism added after each U-Net convolution layer, the SE-ResU-Net model with SENet added after each ResU-Net convolution layer, the CBAM-ResU-Net model with CBAM added after each ResU-Net convolution layer, and the ECA-ResU-Net model with ECA added after each ResU-Net convolution layer are investigated herein. The segmentation results are shown in Fig 13. It can be seen that the segmentation performance of the ECA-ResU-Net model is the best among other models optimized with attention modules.

The evaluation results for different models are shown in Table 1. It is shown that, compared to the traditional U-Net model added with an attention mechanism, the ECA-ResU-Net model with the ECA module has better segmentation performance for blasting fragment images.

**Table 1:** Performance of modified models.

	ACC	AUC	PPV	TPR
**SE-U-Net**	0.9318	0.9705	0.9578	0.9670
**CBAM-U-Net**	0.9340	0.9664	0.9401	0.9544
**ECA-U-Net**	0.9326	0.9721	0.9590	0.9533
**SE-ResU-Net**	0.9492	0.9802	**0.9718**	0.9604
**CBAM-ResU-Net**	0.9505	**0.9815**	0.9705	0.9579
**ECA -ResU-Net**	**0.9619**	0.9804	0.9706	**0.9706**

#### 5.2.3 Model performance considering the position of the attention module and focal loss

**Fig 14:**
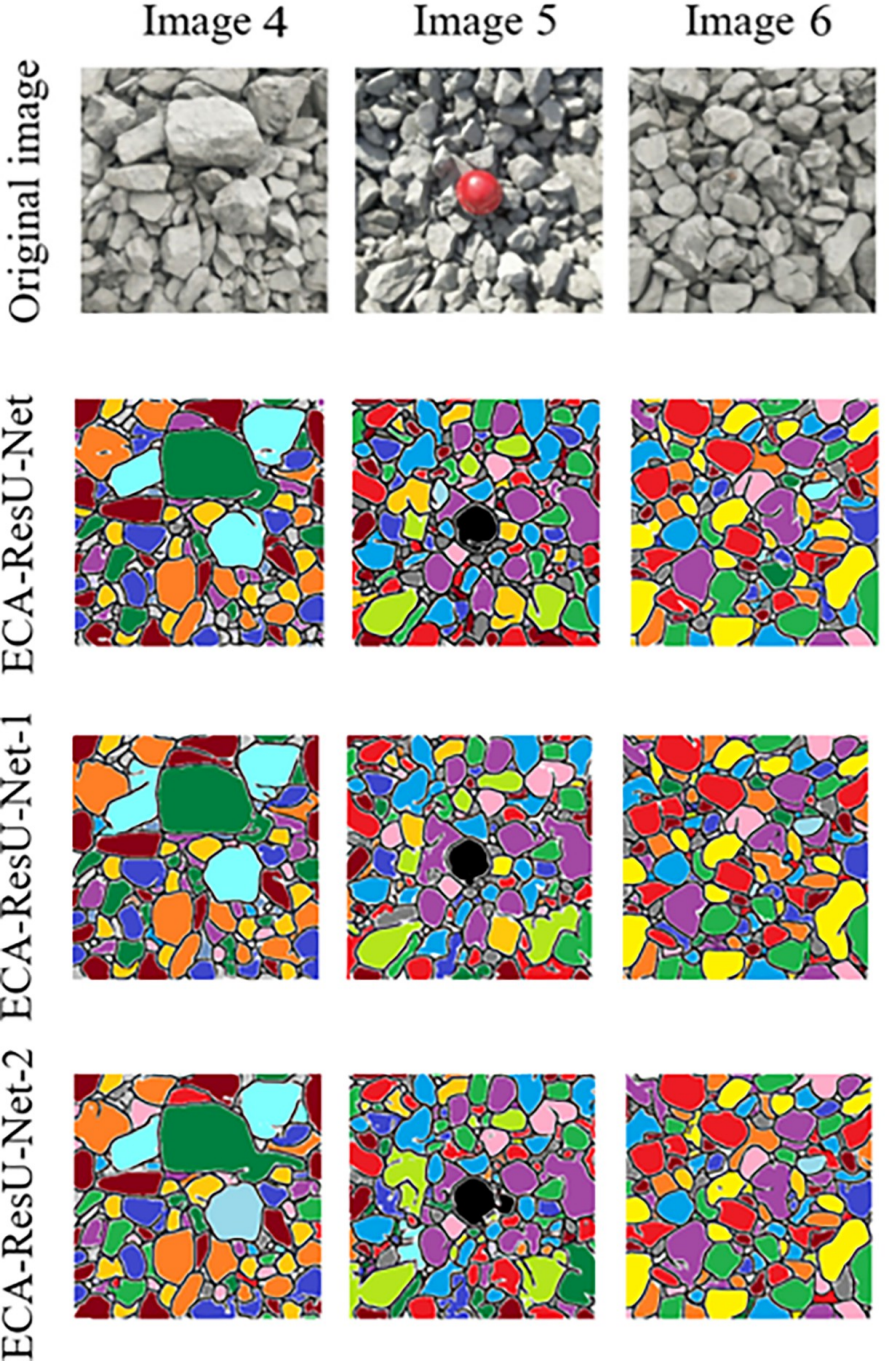
Comparison of the segmentation performance in the models with the varying attention module position.

In order to investigate the effect of the attention module’s position on the training performance, the model is trained multiple times by taking the position of the ECA module as a variable, and the segmentation performance of the model is analyzed. The ECA-ResU-Net model with ECA added after each ResU-Net convolution layer, the ECA-ResU-Net-1 model with ECA added in the down-sampling process, and the ECA-ResU-Net-2 model with ECA added in the up-sampling process are investigated. Finally, the effects of the binary_crossentropy loss function and focal loss function are compared. The analysis results are shown in Fig 14. It can be seen that the segmentation performance of the ECA-ResU-Net-1 model is the best among the other models optimized with attention modules.

In addition, the evaluation results are shown in Table 2. It can be seen that the ECA-ResU-Net-1 model with ECA added in the down-sampling process has better segmentation performance than the models with ECA added in the up-sampling process or the entire process. The results indicate that the attention module’s position impacts the models’ training performance.

**Table 2:** Performance of models with the varying position of the attention module.

	ACC	AUC	PPV	TPR
**ECA-ResU-Net**	0.9619	0.9804	0.9706	0.9706
**ECA-ResU-Net-1**	**0.9662**	**0.9919**	**0.9814**	**0.9789**
**ECA-ResU-Net-2**	0.9641	0.9914	0.9805	0.9774

**Fig 15:**
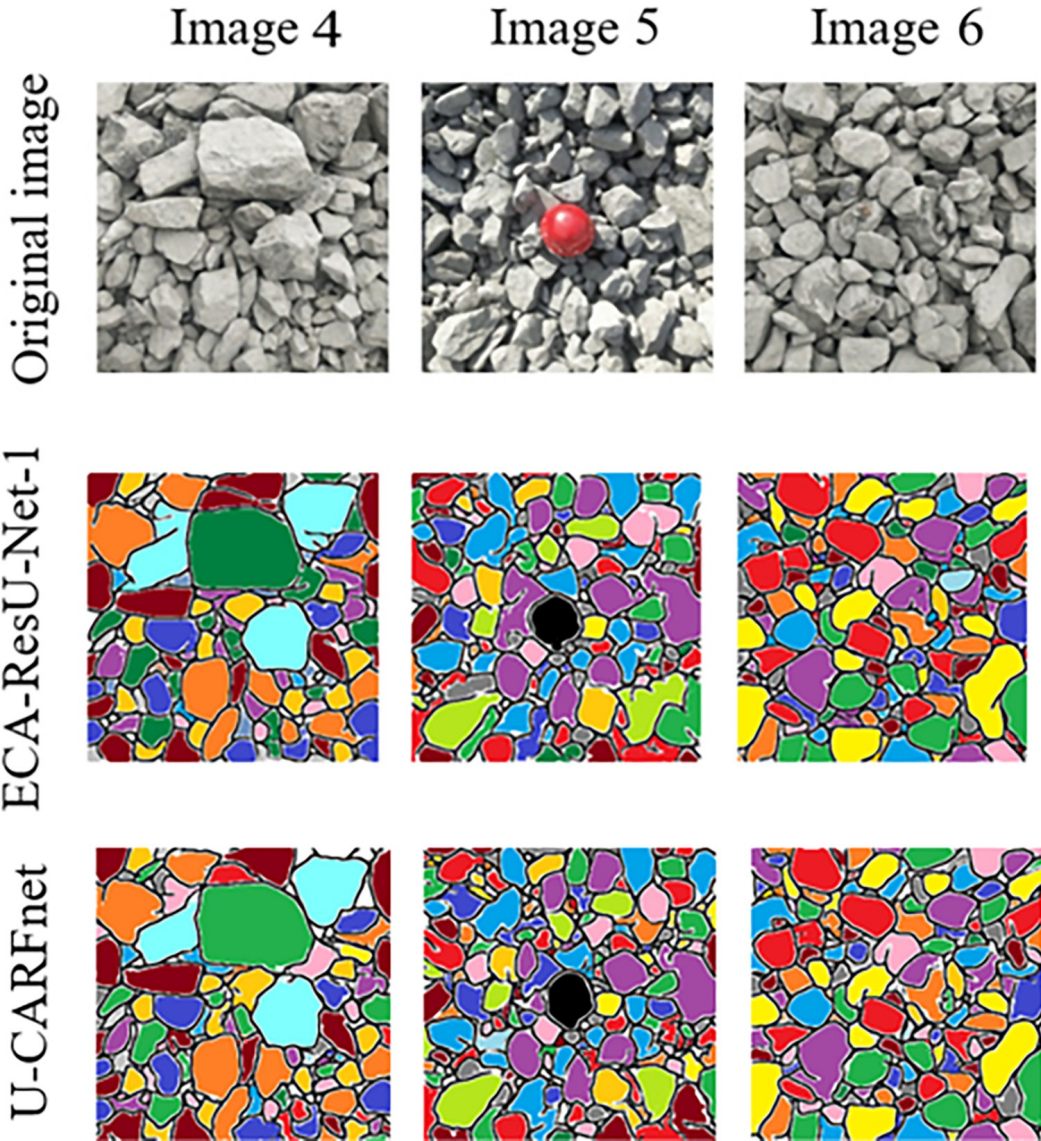
Comparison of the segmentation performance in the optimized models.

Based on the above research, the focal loss function is introduced in the model to improve the segmentation performance. The model training results with the focal loss function and the original loss function, the binary_crossentropy, are compared, as shown in Table 3. The ECA-ResU-Net-1 combined with the focal loss function is defined as the U-CARFnet model. Table 3 and Fig 15 show that most ore fragments are entirely separated, and the segmentation performance is satisfactory. The U-CARFnet model proposed in this paper can achieve higher segmentation precision for blasting fragment images.

**Table 3:** Performance of the optimized models.

	ACC	AUC	PPV	TPR
**ECA-ResU-Net-1**	0.9662	0.9919	0.9814	0.9789
**U-CARFnet**	**0.9711**	**0.9923**	**0.9820**	**0.9814**

#### 5.2.4 Comparison with other models

Currently, the methods based on the lumpiness distribution’s statistics for ore and rock mainly require manual calculation, traditional image segmentation methods (such as edge detection (Canny [26])), some granularity statistics software, or a small amount of rock image segmentation combined with a deep learning method. Among them, the manual calculation method is influenced by human factors, and in the case of a large ore block area, it can only be sampled statistically, its accuracy is questionable, and it requires huge computational efforts. The traditional image segmentation method has poor segmentation capability, with many over-segmentation and under-segmentation problems. For example, the WipFrag granularity statistics software (Maerz et al. [27]) takes a certain period to manually clean the segmentation lines generated during the statistics of the exploded heap’s fragmentation. With the increase in the number of processed images, the processing time is increased rapidly. Some deep learning methods only study the images of rock and ore on the conveyor belt, and there are few studies on the lumpiness of rock and ore in open pit mines.

**Fig 16:**
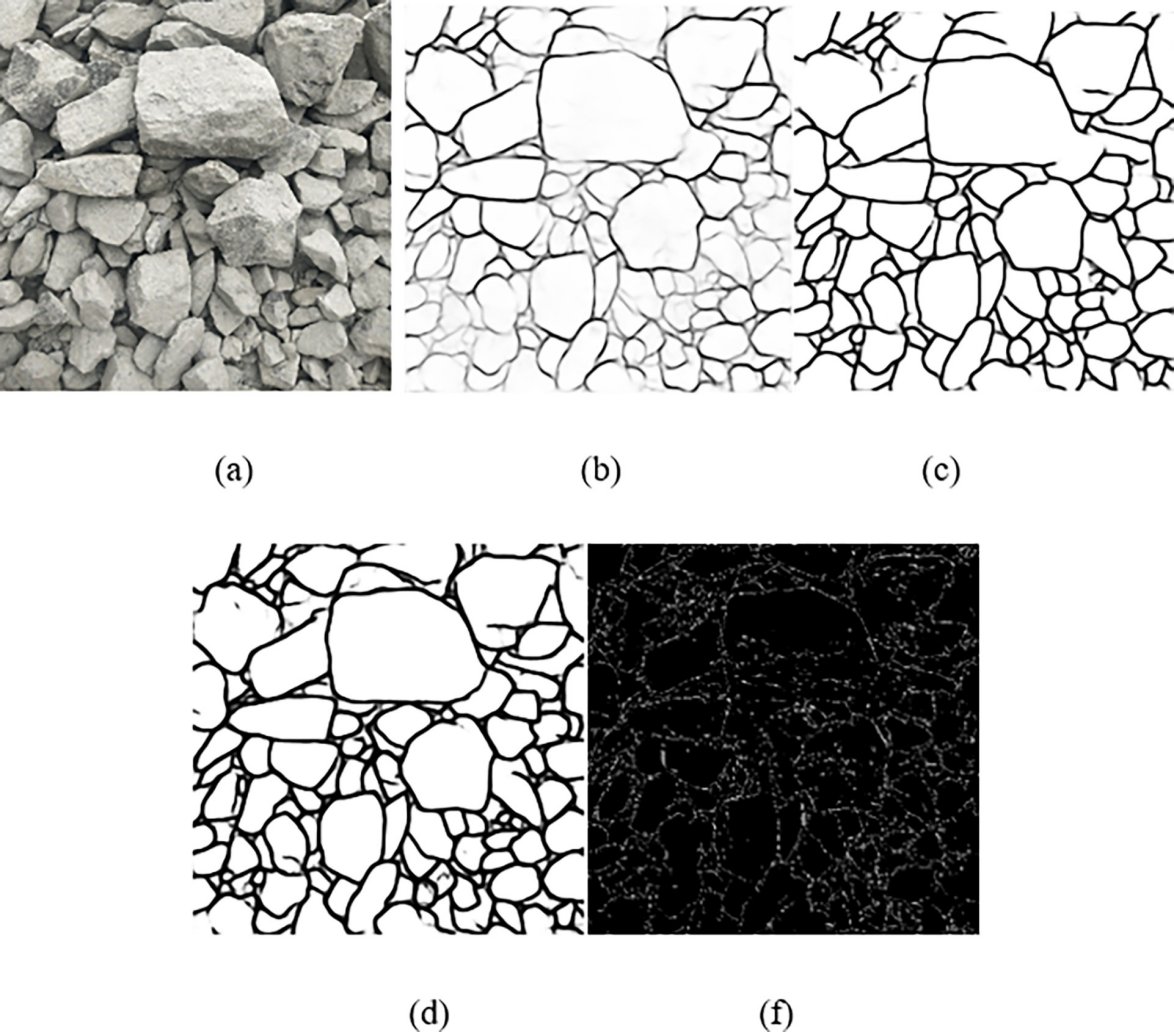
Comparison of segmentation performance. (a) Original image (b) Segmentation by U-Net model (c) Segmentation by ResU-Net model (d) Segmentation by U-CARFnet (f) Edge detection effect based on Canny operator.

In order to verify the effectiveness of the proposed U-CARFnet image segmentation model in an open-pit mine explosion, the model was compared to the repeated U-Net and ResU-Net deep learning models and the traditional image segmentation method (edge detection based on the Canny operator). It can be seen from Table 4 and Fig 16 that the U-Net model, ResU-Net model, and traditional image segmentation methods all have many under-segmentation and over-segmentation problems in the segmentation effect of blasting rock images. Experimental results show that the accuracy of the u-CARFnet model proposed in this paper reaches 97.11% in the performance evaluation, which shows better performance than the traditional image segmentation method.

**Table 4:** Comparison of the U-Net, ResU-Net, and U-CARFnet performance.

	ACC	AUC	PPV	TPR
**U-Net**	0.9263	0.9445	0.9556	0.9604
**ResU-Net**	0.9577	0.9719	0.9603	0.9714
**U-CARFnet**	0.9711	0.9923	0.9820	0.9814

### 5.3 Application of U-CARFnet

In order to validate the segmentation performance of the U-CARFnet for blasting fragment images, the image processing technique, OpenCV, is employed to monitor the number of ore fragments in the predicted contour image. The ratio between the number of ore fragments in the segmented image and the total number of ore fragments are defined as accuracy (A). The error rate (E) is the ratio between the number of unsegmented and falsely segmented ore fragments and the total number of ore fragments. The *C*_*nb*_ is the number of accurately recognized ore fragments, *W*_*nb*_ is the number of unrecognized ore fragments, and MC is the manual calculation result. Table 5 compares the recognition results of the U-Net and U-CARFnet models. The average error of segmentation by the U-CARFnet model is 5.46%, which is better than that of the U-Net model and can satisfy mining applications.


$$A=\frac{C_{nb}}{C_{nb}+W_{nb}}$$ (11)

$$E=\frac{W_{nb}}{C_{nb}+W_{nb}}$$ (12)

**Table 5:** Comparison of contour extraction for blasting fragment images by U-Net and U-CARFnet model.

Model	Image	*C*_*nb*_	*W*_*nb*_	MC	E(%)
U-Net	Image4	87	22	109	20.18
	Image5	93	29	122	23.77
	Image6	93	26	119	21.85
	Mean				21.93
U-CARFnet	Image4	102	7	109	6.42
	Image5	116	6	122	4.92
	Image6	113	6	119	5.04
	Mean				5.46

In addition, the time required to infer results in the U-CARFnet model is determined and compared with the manual calculation. Generally, it only takes 1 to 2 seconds on average for the U-CARFnet model to infer the number of ore particles in a blasting fragment image, which is significantly lower than manual calculation. Furthermore, the testing images in Figs 15 and 16, which differ from the training images, show that most ore fragments are segmented with good performance. This indicates that the proposed model has a good generalization capability and can be applied to segment other blasting fragments images. At the same time, when the original image is input into WipFrag granularity statistical software, the post-processing takes a long duration. However, the segmentation result image obtained through the U-CARFnet model is then input into the software for the statistics of rock fragmentation distribution, which can eliminate the post-processing time and significantly improve the use efficiency.

## 6. Conclusions

This paper proposes a U-CARFnet model to segment blasting fragment images. The attention mechanism and the residual learning module are integrated into the model to optimize the U-Net. Generally, the study results showed that the model performs well in segmenting ore fragment images from bench blasting in open-pit mines. Moreover, the U-CARFnet model proposed in this paper achieves the best segmentation effect, which shows an accuracy of 97.11% in the performance evaluation and gives a superior performance with an average segmentation error of 5.46% in application. The segmentation results of the U-CARFNET model can be used in some granularity statistics software to improve the statistical efficiency of rock fragmentation distribution and facilitate studies on blasting fragmentation prediction.

## Abbreviations

HSV: HSV(Hue, Saturation, Value), also known as Hexcone Model, is A color space created by A. R. Smith in 1978 according to the intuitive characteristics of colors. HSV color model refers to a visible light subset in the three-dimensional color space of H, S and V, which contains all the colors of a certain color domain. HSV(Hue, Saturation, Value), also known as Hexcone Model, is A color space created by A. R. Smith in 1978 according to the intuitive characteristics of colors. HSV color model refers to a visible light subset in the three-dimensional color space of H, S and V, which contains all the colors of a certain color domain
CBAM: Convolutional Block Attention Module
ECA: Efficient Channel Attention
